# Models of Early Resistance to CDK4/6 Inhibitors Unveil Potential Therapeutic Treatment Sequencing

**DOI:** 10.3390/ijms26062643

**Published:** 2025-03-14

**Authors:** Elisabet Zapatero-Solana, Yan Ding, Nicholas Pulliam, Alfonso de Dios, Maria Jesus Ortiz-Ruiz, María José Lallena

**Affiliations:** 1 Lilly S.A.U., Cell Biology & Translational, DCRT, 28108 Madrid, Spain; zapatero_elisabet@lilly.com; 2 Eli Lilly and Company, Indianapolis, IN 46285, USA; yan.ding@lilly.com (Y.D.); npulliam@lilly.com (N.P.); de_dios_alfonso@lilly.com (A.d.D.)

**Keywords:** breast cancer, CDK4/6 inhibitor, abemaciclib, endocrine therapy, resistance

## Abstract

Background: CDK4/6 inhibitors (CDK4/6i) combined with hormone therapies have demonstrated clinical benefit in HR+, HER2- breast cancer patients. However, the onset of resistance remains a concern and highlights a need for therapeutic strategies to improve outcomes. The objective of this study was to develop an in vitro model to better understand the mechanisms of resistance to CDK4/6i + hormone therapies and identify therapeutic strategies with potential to overcome this resistance. Methods: The HR+, HER2− T47D breast cancer cell line genetically modified with a Geminin–Venus reporter construct was treated with CDK4/6i (abemaciclib or palbociclib) in combination with 4-hydroxytamoxifen (tamoxifen). Resistant cells were identified by cell sorting for Geminin (%GEM+), a marker of the S/G2/M phases of the cell cycle, and confirmed by treatment with tamoxifen plus the CDK4/6i used to drive resistance. In resistant cells, following treatment with CDK4/6i + ET (tamoxifen or fulvestrant), the effects on cell proliferation (%GEM+) and viability, gene expression, and protein analysis to evaluate CDK4/6–cyclin D complex composition were examined. Results: Palbociclib + tamoxifen-resistant (PTxR) cells treated with abemaciclib + ET showed decreased %GEM+, %Ki67, and colony formation ability, compared to abemaciclib + tamoxifen-resistant (ATxR) cells treated with palbociclib + ET. Additionally, PTxR cells showed increased CDK4-p21 interaction, compared to ATxR. The CDK6 levels were greater in ATxR cells compared to PTxR cells, associated with CDK4/6i resistance. Additionally, abemaciclib + fulvestrant continued to robustly decrease pRb levels in PTxR models compared to palbociclib + fulvestrant in ATxR models. Transcriptome analysis revealed a depression of the cell cycle and E2F- and Rb-related genes in PTxR cells following treatment with abemaciclib + ET, not present in ATxR cells treated with palbociclib + ET. Both resistant models showed increased EGFR-related gene expression. Conclusion: Taken together, we describe CDK4/6i-dependent mechanisms resulting in early-onset resistance to CDK4/6i + ET, using clinically relevant drug concentrations, in preclinical breast cancer cell models. The characterization of these preclinical models post progression on CDK4/6 inhibitor + ET treatment highlights the potential that the specific sequencing of CDK4/6 inhibitors could offer to overcome acquired resistance to CDK4/6i + ET. Abemaciclib + fulvestrant is currently under clinical investigation in patients with HR+, HER2− breast cancer and progression on prior CDK4/6i + ET (NCT05169567, postMONARCH).

## 1. Introduction

Breast cancer remains the most common cancer among women worldwide and a leading cause of cancer death in women [1]. Of breast cancer diagnoses, hormone receptor-positive (HR+), human epidermal growth factor receptor 2-negative (HER2−) is the most common subtype, representing approximately 70% of all breast cancer diagnoses [2]. Several studies have identified a role for cyclin-dependent kinases 4 and 6 (CDK4/6) as actionable therapeutic targets in combination with fulvestrant, resulting in the approval of CDK4/6 inhibitors (CDK4/6i) + fulvestrant as the clinical standard of care [3,4,5,6,7,8]. Despite these milestones, resistance to combination CDK4/6 inhibitors plus ET remains a concern and is currently under investigation in the phase 3 postMONARCH clinical trial [9,10].

The postMONARCH study, a phase 3, randomized, placebo-controlled trial, demonstrated that switching to abemaciclib plus fulvestrant significantly reduced the risk of disease progression in HR+, HER2− advanced breast cancer with progression on prior CDK4/6i plus endocrine therapy. Given the clinical relevance and benefit of abemaciclib + fulvestrant post progression on prior CDK4/6i + ET, a better understanding of the molecular characteristics and potential mechanisms contributing to the onset of resistance to CDK4/6i + ET and therapeutic strategies to overcome this resistance is of great interest [11,12].

Several mechanisms leading to adaptation and resistance to CDK4/6i have been identified [13,14,15], including CDK6 overexpression [16], *RB1* loss [17], compensation through the bypass of CDK4 signaling, and the increased expression of CDK2 [18,19]. Furthermore, a recent study demonstrated that the oxidative phosphorylation pathway may also play a role in mediating resistance to palbociclib. Interestingly, this mechanism was not present in abemaciclib-resistant models [20], suggesting that CDK4/6i therapeutic resistance may be drug-dependent. To note, the concentration of CDK4/6i used in this study to drive resistance may lack clinical relevance [20].

A major limitation in understanding the mechanisms behind early-onset therapeutic resistance to combination CDK4/6i + ET is the lack of available in vitro models [21,22]. Early-onset resistance is canonically intrinsic to dividing cells (S/G2/M), rather than quiescent cells, which are resistant to therapies that target cells undergoing cell division [21]. The fluorescence ubiquitination cell cycle indicator (FUCCI) system utilizes fluorescent reporter genes that target and allow for the identification of cells in specific cell cycle phases [23]. We treated Geminin–Venus reporter-expressing breast cancer cells with clinically relevant concentrations of abemaciclib + tamoxifen or palbociclib + tamoxifen [24]. After treatment, cells were sorted to identify those populations that continued to proliferate (GEM+) in the presence of the drug. Given the cytostatic effects of CDK4/6 inhibitors [13], cells that continued to proliferate were classified as the resistant population. To confirm therapeutic resistance in this population, cells were treated with clinically relevant doses of the respective CDK4/6 inhibitor used to drive resistance, along with tamoxifen, and cell proliferation was measured (Supplemental Figure S1).

Following the establishment of resistance, we showed that palbociclib + tamoxifen-resistant (PTxR) cells treated with abemaciclib + ET (fulvestrant or tamoxifen) had decreased %GEM+, %Ki67, and cell proliferation. The effects on %GEM+ and cell proliferation were significantly greater following treatment with abemaciclib + fulvestrant and not observed in ATxR cells treated with either ribociclib or palbociclib + ET. These data demonstrate that PTxR cells continue to respond to abemaciclib + ET, whereas ATxR cells are cross-resistant to palbociclib + ET. Further, we show that the composition of the CDK4/6 complex and its interaction with p21 or p27 may represent a biomarker of continued CDK4/6i + ET response following the gain of resistance. For the first time, we elucidate the molecular characteristics of early adaptation and resistance to CDK4/6i + ET, using clinically relevant drug concentrations, in HR+, HER2− breast cancer and identify therapeutic strategies to overcome acquired resistance in this setting.

## 2. Results

### 2.1. Early Resistance to CDK4/6 Inhibitor Plus Tamoxifen

To initially understand the mechanisms of early resistance to CDK4/6i + ET, the HR+, HER2− T47D breast cancer cell line was selected. Following the generation of T47D cells constitutively expressing the Geminin + Venus reporter (active S/G2/M phase, Section 4), T47D GEM+ cells were treated with clinically relevant concentrations of either abemaciclib plus tamoxifen or palbociclib plus tamoxifen for 48–336 h (Supplemental Table S1 and Figure S1). Treated cells were collected and analyzed by fluorescence-activated cell sorting (FACS). Between 120 and 144 h of treatment, the %GEM+ cell population rebounded and was near steady state, suggesting treatment resistance (Supplemental Figure S1A,B). To confirm the onset of resistance in the resultant GEM+ population, ATxR and PTxR cells were maintained in tamoxifen plus the CDK4/6 inhibitor used to drive resistance, and the %GEM+ population was measured. In both ATxR and PTxR cells, %GEM+ was approximately 10% and 11%, respectively (Supplemental Figure S1A,B).

### 2.2. Switch to Abemaciclib + ET Resensitizes Cells to CDK4/6i Following Progression on Palbociclib + Tamoxifen

**Figure 1:**
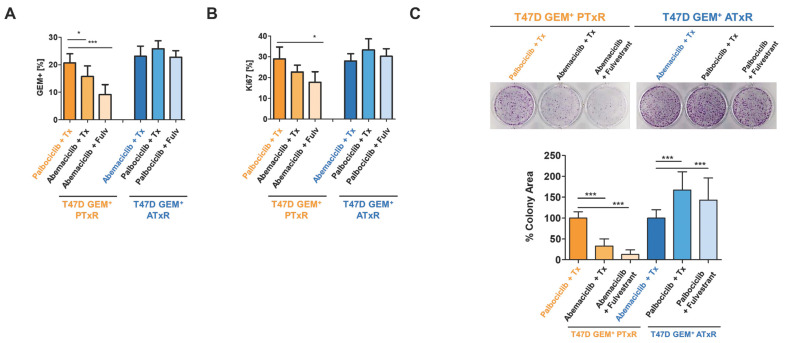
Sequential treatments in T47D GEM+ PTxR and T47D GEM+ ATxR cells (2nd treatment). (**A**) GEM+% and (**B**) Ki67% were measured in parallel to validate Geminin as an alternative proliferation marker to the standard Ki67 to assess the proliferating fraction of a cell population (n = 3, two-tailed, unpaired Student’s *t*-test to resistant control line). (**C**) Colony formation assay in T47D GEM+ PTxR and T47D GEM+ ATxR cell lines. In total, 10,000 cells/well were seeded and cultivated in the presence of the secondary treatments, as well as vehicle control (DMSO/methanol) for 14 days. Colonies were fixed using 100% methanol for 5 min RT and stained with 0.1% crystal violet for 1–2 min. Colony area was quantified with Image J (n = 3, two-tailed, unpaired Student’s *t*-test to resistant control line). Representative image is shown for n = 3. * *p* < 0.05, *** *p* < 0.001.

Next, to investigate if resistance to CDK4/6i + tamoxifen altered the response to CDK4/6i generally, we treated ATxR cells with palbociclib + ET (tamoxifen or fulvestrant) and PTxR cells with abemaciclib + ET and measured the %GEM population (Supplemental Figure S2). In PTxR cells treated with abemaciclib + ET, we observed decreased (*p* < 0.05) %GEM+ cells, indicating decreased cell proliferation (Figure 1A). This effect was greater following the switch to abemaciclib + fulvestrant, compared to tamoxifen. Conversely, ATxR cells treated with palbociclib + ET showed no difference (*p* > 0.05) in the %GEM population (Figure 1A). Given the relevance of Ki-67 as an established biomarker for breast cancer prognosis and cell proliferation [25,26], we next measured %Ki-67 in these models before and after the therapeutic switch. Similar to the %GEM, we observed decreased (*p* < 0.05) %Ki-67 in PTxR cells treated with abemaciclib + fulvestrant. This effect was not observed in ATxR cells treated with palbociclib + ET (Figure 1B). While the therapeutic switch of ATxR cells to palbociclib + ET was not sufficient to overcome resistance to CDK4/6 inhibitors, we examined whether ribociclib + ET, another clinically available CDK4/6i, was sufficient to decrease the %GEM and %Ki-67 population in our resistant models. PTxR and ATxR cells were treated with ribociclib + ET following the same schema as for abemaciblib and palbociclib (Supplemental Figure S2), and %GEM and %Ki-67 were measured. As per PTxR cells treated with abemaciclib + ET, ribociclib + ET decreased (*p* < 0.05) both the %GEM and %Ki-67 population, but a non-statistically significant trend in activity was observed in ATxR cells (Supplemental Figure S3A,B).

To validate these results, we performed colony formation and apoptosis assays under the same conditions. In PTxR cells, following treatment with either abemaciclib or ribociclib + ET, we observed decreased (*p* < 0.05) cell proliferation, indicated by a decreased % colony area (Figure 1C, Supplemental Figure S3C). This effect was greater following treatment with either abemaciclib or ribociclib + fulvestrant, compared to tamoxifen. In ATxR cells, following the therapeutic switch to palbociclib + ET, we observed increased (*p* < 0.05) cell proliferation regardless of the ET used (Figure 1C). In contrast, ribociclib + ET decreased (*p* < 0.05) the % colony area (Supplemental Figure S3C). Similarly, we observed robustly increased (*p* < 0.05) apoptosis in PTxR cells following treatment with abemaciblib and a modest increase in apoptosis in either PTxR or ATxR cells following treatment with ribociclib + ET (Supplemental Figure S4A,B). Together, our data indicate that PTxR cells have both a cytostatic and cytotoxic response to sequential CDK4/6 inhibitor + ET treatment and that ATxR cells remain unresponsive to palbociclib and modestly responsive to ribociclib + ET.

### 2.3. Biomarker Modulation

**Figure 2:**
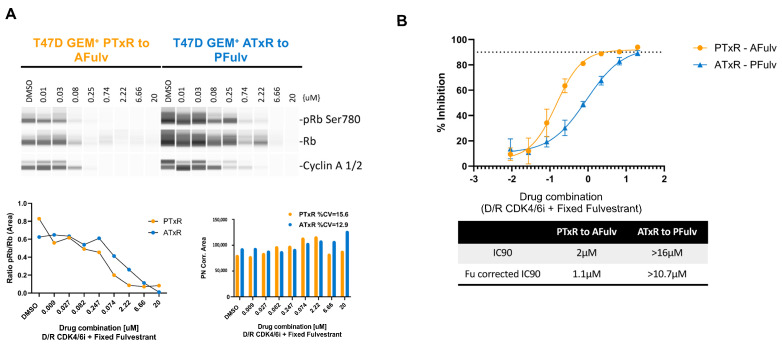
Biomarker evaluation in resistant cell lines. (**A**) Biomarker modulation and (**B**) % inhibition were measured to examine whether the lack of sensitivity of ATxR cells to palbociclib was due to the level of biomarker modulation. Efficacy of CDK4/6 inhibitors was measured in a dose–response assay with a fixed concentration of fulvestrant (data are shown as the average of two biological replicates). % Inhibition was calculated as 100—normalized GEM+ treated cells to vehicle. Protein normalization assay was used to demonstrate robust protein content across all samples.

Canonically, CDK4 and CDK6 regulate the tumor suppressor retinoblastoma (Rb) protein [27] through direct phosphorylation (pRb) [27,28]. The loss of CDK4/6-mediated Rb regulation, resulting in increased pRb, is associated with the onset of CDK4/6i resistance. We performed a dose–response analysis of Rb and pRb protein expression following the treatment of both PTxR and ATxR cells with increasing concentrations of the opposing CDK4/6 inhibitor (abemaciclib or palbociclib) plus a fixed concentration of fulvestrant, the ET of choice in postMONARCH [10]. The treatment of PTxR cells with abemaciclib + fulvestrant resulted in a near-complete depletion of pRb protein expression at a concentration of 250 nM abemaciclib (Figure 2A). In contrast, ATxR cells required a higher concentration of palbociclib (2.2 µM) + fulvestrant to achieve a similar depletion of pRb expression (Figure 2A). Additionally, the total Rb levels were more substantially decreased in PTxR cells treated with abemaciclib + fulvestrant compared to ATxR cells treated with palbociclib + fulvestrant. This suggests that the reduction in pRb levels may be due to both Rb degradation and the inhibition of CDK4/6. The cyclin A levels were diminished similarly to those of pRb (Figure 2A). To further validate these findings, we measured the % inhibition of PTxR and ATxR cells treated as in the prior experiment with the opposing CDK4/6i + fulvestrant. The Fu-corrected IC_90_ of PTxR cells treated with abemaciclib + fulvestrant was 1.1 µM, whereas for ATxR cells treated with palbociclib + fulvestrant, the Fu-corrected IC_90_ was >10.7 µM (Figure 2B). This trend was consistent with the pRb dose–response analysis. The IC_90_ values were corrected to account for the fraction unbound (Fu) for comparison purposes. This adjustment ensures that we are considering only the portion of each compound that is not bound to proteins, as this unbound fraction is expected to have a pharmacological effect. Overall, these data suggest that abemaciclib continues to robustly inhibit CDK4/6-mediated Rb phosphorylation and promote total Rb degradation in PTxR cells, an effect not observed with palbociclib in ATxR models.

In addition to the regulation of the CDK4/6-Rb signaling axis, a recent study suggested that CDK4/6 inhibitor efficacy is determined in part through off-target effects driven by CDK9 signaling and G2/M progression [29]. To note, the concentrations of CDK4/6 inhibitor used in this study may lack clinical relevance. In our study, Western blot analysis of CDK9-associated proteins showed a lack of inhibition of RNA POLII (p-Ser2; C-terminal domain) and MCL1 following treatment with either abemaciclib or palbociclib + ET (Supplemental Figure S5). Additionally, in ATxR cells, following the therapeutic switch, there was no change in pHistone H3-Ser10, Wee1, CDC2, or topoisomerase II. These data suggest that CDK9-related off-target effects do not mediate CDK4/6i efficacy in our models, and cells do not undergo G2/M arrest.

### 2.4. coIPs and Jess Analysis

**Figure 3:**
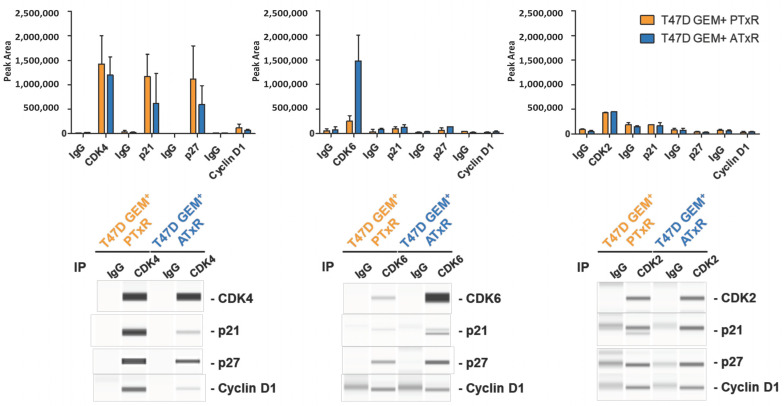
Co-immunoprecipitation (Co-IP) and Jess immunoblot analysis of p21/p27–cyclin D1–CDK4, p21/p27–cyclin D1–CDK6, and p21/p27–cyclin D1–CDK2 complexes in resistant lines. Fresh protein lysates of PTxR and ATxR cells were immunoprecipitated with either anti-CDK4, anti-CDK6, or anti-CDK2 antibodies and immunoblotted with anti-cyclin D1, p21, and p27 by Jess. Quantification of total peak area to determine levels of protein and Western blot-like images are shown. Data are represented as the average of two biological replicates. The control was normal rabbit serum.

The protein expression of CDK6 or CDK2 compared to CDK4 represents known mechanisms of resistance to CDK4/6 inhibitors [18]. Additionally, recent studies have demonstrated that the composition of the CDK4–cyclin D1 complex and its interaction with p21 and p27 alter the response to CDK4/6 inhibitor treatment [18,30]. To examine the composition of the CDK4, CDK6, or CDK2 complex in association with cyclin D1 in ATxR and PTxR cells, we immunoprecipitated (IP) the indicated proteins and measured their interaction with p21, p27, and cyclin D1. In both PTxR and ATxR cells, we observed similar levels of CDK4 and CDK2 and increased CDK6 levels in ATxR cells compared to in PTxR cells (Figure 3). Furthermore, in PTxR cells, CDK4-p21 was greater compared to in ATxR cells. Following the immunoprecipitation of CDK6, no changes in interacting proteins were observed in either PTxR or ATxR cells. CDK4-p21 interaction has been associated with an increased response to CDK4/6 inhibitors compared to CDK4/6-p27 interaction [18,30]. No differences were observed in CDK2-p21/p27 interaction in either ATxR or PTxR cells (Figure 3). These results corroborate our previous observations (Figure 2) and suggest CDK4/6 inhibitor-dependent mechanisms of resistance. These results are consistent with the differential response of ATxR and PTxR cells to the therapeutic switch and support the hypothesis that the composition and expression of the CDK4/6 complex may be indicative of the therapeutic response to CDK4/6 inhibitors.

### 2.5. Molecular Characterization of Resistant Cell Lines Following Therapeutic Switch

**Figure 4:**
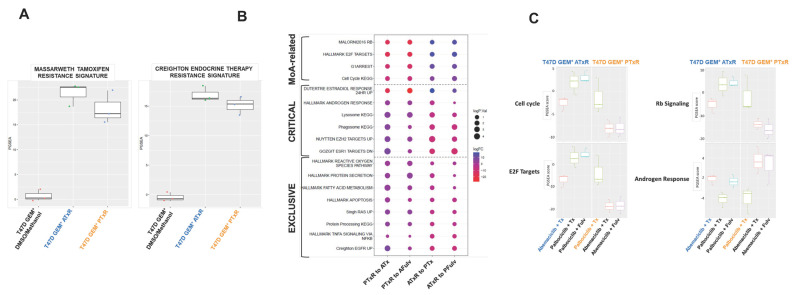
Pathway analysis for RNAseq data from T47D GEM+ PTxR and ATxR cell lines upon treatment switch. RNAseq was performed on an Illumina NovaSeq 6000 using the Illumina TruSeq Stranded mRNA Library Prep Kit. The gene set (pathway) definitions were either generated from the KEGG official website or downloaded from the Molecular Signature Database. Parameter gene set enrichment analysis (PGSEA) 2 was used to generate enrichment scores for each pathway within each sample using the vehicle control cell lines (for ATxR cell line using abemaciclib/Tx treatment; for PTxR cell line using palbociclib/Tx treatment) as references. A moderated *t*-statistic implemented in the limma package was used to identify gene set enrichment scores that could discriminate between groups. (**A**) An increase in tamoxifen and ET resistance signatures demonstrates ET resistance for both cell lines. (**B**) Main pathways altered upon treatment switch based on logFC and log *p*-value. (**C**) Box plot representing main proliferation-related signatures (MoA-related) in the different treatment switches, and androgen response was found to be a potential tumor suppressor (n = 3).

Given the differential phenotypic responses of the resistant cell models, we investigated the underlying molecular characteristics after treatment with tamoxifen and the CDK4/6 inhibitor used to drive resistance and following the therapeutic switch. To examine global gene expression changes, RNAseq analysis was performed in ATxR and PTxR cells before and after the therapeutic switch. Multiple pathway algorithms were used to select the top changed gene signatures/pathways using either KEGG, MSIG HALLMARK gene signatures, or internal curated oncogenic gene signatures (Supplemental Table S2). The top altered HALLMARK and oncogene signatures are highlighted as a heatmap in Supplemental Figure S6; based on the cutoff, at least five algorithms’ *p* < 0.01 and fold change in PGSEA scores are either more than 2 or less than -2 and PGSEA *p* < 0.01 (n ≥ 5 and abs(logFC) > 2 and *p* < 0.01). Compared with the T47D GEM+-vehicle-treated cell lines, the endocrine therapy resistance gene signatures were significantly increased in both the ATxR and PTxR cell lines (Figure 4A). The top gene signature/pathway changes, following the therapeutic switch, were highlighted using dot plots and grouped by mechanism of action-related, critical, and exclusive functions (Figure 4B). In PTxR cells, following the therapeutic switch, transcriptome analysis revealed the depression of the cell cycle and E2F- and Rb signaling-related genes and increased androgen response (AR)-related genes (Figure 4C). In ATxR cells, following the therapeutic switch, we observed an increased expression of genes related to Rb signaling and cell cycle regulation and a further depression of AR-related genes. Interestingly, genes related to the androgen response showed increased expression in untreated ATxR cells relative to untreated PTxR cells. The treatment of ATxR cells with palbociclib + tamoxifen resulted in the depression of AR-related gene expression, not observed with fulvestrant treatment. In contrast, PTxR cells treated with abemaciclib + ET exhibited an increased expression of AR-related genes, regardless of the ET used. These results are consistent with our prior observations of PTxR cells continuing to derive benefit from abemaciclib + ET and highlight recent reports which have identified both a potential tumor suppressive and oncogenic role for the androgen receptor in ER+ breast cancer [31,32,33].

**Figure 5:**
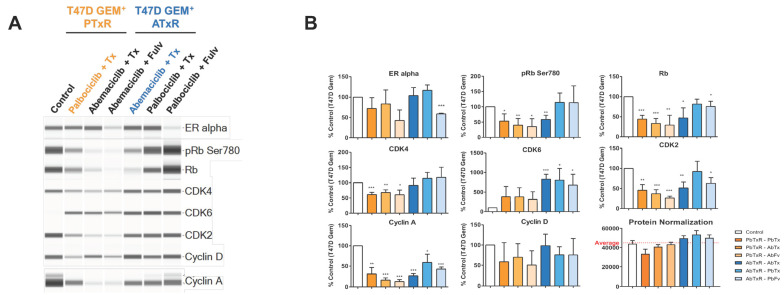
Analyses of the protein levels of ER, pRb Ser780, Rb, CDK4, CDK6, CDK2, cyclin D1, and cyclin A1/2 1 in resistant PTxR and ATxR cell lines and drug switches. (**A**) Western blot-like images of cell cycle marker panel and (**B**) quantification of total peak area to determine changes in target protein expression, represented as % control calculated with T47D Geminin parental cell line treated with DMSO/methanol (n = 3, two-tailed, unpaired Student’s *t*-test to vehicle control). Due to the modulation of standard housekeeping proteins (tubulin and vinculin) by the different treatments used, a protein normalization assay was used to demonstrate robust protein content across all samples. * *p* < 0.05, ** *p* < 0.01, *** *p* < 0.001.

In the protein analysis, compared to the control, baseline ATxR and PTxR cells continued to express ERα, and pERK1/2 levels were increased (*p* < 0.05), previously described in models of tamoxifen resistance and in line with our endocrine-resistant gene signature (Figure 5 and Figure S7A; refs. [34,35]). Consistent with the onset of CDK4/6i resistance, CDK6 levels were increased (ATxR: *p* < 0.05) relative to the control [17,36]. Interestingly, the magnitude of FoxM1, CDK2, and cyclin D expression was lower in PTxR cells, compared to in ATxR cells (Figure 5 and Figure S7A,B), previously demonstrated to mediate the response to CDK4/6i treatment [18,37]. In PTxR cells, following the therapeutic switch, we observed decreased (*p* < 0.05) Rb, pRb, cyclin A, and CDK2 protein levels, and following treatment with abemaciclib + fulvestrant only, pERK1/2 and ERα levels were also decreased (*p* < 0.05). In ATxR cells, the switch to palbociclib + ET resulted in increased (*p* < 0.05) pRb levels (Figure 5A). Interestingly, we observed an increase in EGFR-related genes in ATxR and PTxR cells. Overall, these results suggest continued Rb/E2F signaling in ATxR cells treated with palbociclib + ET, consistent with our prior observation (Figure 2).

## 3. Discussion

The majority of advanced or metastatic breast cancer (ABC) patients are diagnosed with HR+, HER2− disease [1,2], and the introduction of CDK4/6 inhibitors in combination with endocrine therapies has significantly improved patient outcomes in this setting [3,4,38,39,40]. In nextMONARCH, abemaciclib plus tamoxifen demonstrated significantly improved overall survival (24.2 vs. 17.0 months; *p* = 0.03) [39] and numerically improved PFS (9.1 vs. 7.4 months) in patients with heavily pretreated endocrine-refractory disease compared to abemaciclib alone [39,40]. Similarly, MONALEESA-7 demonstrated a statistical improvement in the 42-month overall survival (70.2% vs. 54.5%; HR for death, 0.71; 95% CI [0.54, 0.95] *p* = 0.00973) and median PFS (22.1 vs. 11.0 months; HR 0.59 95%CI [0.39, 0.88], *p* = 0.03) in patients treated with ribociclib plus tamoxifen compared to tamoxifen alone [41,42]. It should be noted that the control arms differed between nextMONARCH (abemaciclib alone) and MONALEESA-7 (tamoxifen alone). Despite these clinical milestones, early adaptation and resistance to CDK4/6i + ET remains a concern. The postMONARCH trial is a global, phase 3, randomized, placebo-controlled study designed to evaluate the efficacy of abemaciclib plus fulvestrant in patients with HR+, HER2− advanced breast cancer and disease progression on prior CDK4/6 inhibitor plus endocrine therapy (NCT05169567). The trial demonstrated that abemaciclib plus fulvestrant significantly reduced the risk of disease progression compared to placebo plus fulvestrant, with a hazard ratio (HR) of 0.73 (95% CI, 0.57–0.95) [43]. These findings highlight the potential benefits of abemaciclib + fulvestrant beyond disease progression on prior CDK4/6i + ET, offering a targeted therapy option for patients [43,44]. However, there are few in vitro models to understand this occurrence and the mechanisms involved. Additionally, studying early-onset resistance can provide insights into the advantages of continued CDK4/6i + ET treatment in patients who exhibit a short response to first-line CDK4/6i + ET [45]. A notable limitation is the challenge in distinguishing between cells with primary (intrinsic) versus fully acquired resistance, given the timeline utilized to identify resistant cells, though they are treated prior to selection.

Aligning with the postMONARCH inclusion criteria (progression on CDK4/6i and eligible to receive subsequent ET) [10], we developed CDK4/6i (abemaciclib or palbociclib) + ET (tamoxifen)-resistant cell lines utilizing clinically relevant concentrations of either abemaciclib or palbociclib plus tamoxifen. Given the goal to study early-onset resistance to CDK4/6i + ET, tamoxifen rather than aromatase inhibitors [46] were used to develop resistant cell lines. Aromatase inhibitors often take longer to induce resistance due to the gradual depletion of estrogen and the subsequent activation of alternative signaling pathways. This extended timeline can complicate the study of early resistance mechanisms and delay the identification of resistant cell populations.

Cells no longer responding to the CDK4/6i used to drive resistance + ET were denoted as the resistant population. Our study demonstrates that, following treatment with clinically relevant unbound concentrations of abemaciclib + ET, cells resistant to palbociclib + tamoxifen (PTxR) exhibited both cytostatic and cytotoxic responses in vitro. We observed significantly decreased colony formation ability, cell proliferation, %GEM+ population, and Ki-67 levels. Conversely, the treatment of ATxR cells with palbociclib + ET did not affect the colony-forming ability, %GEM+ population, or Ki-67 levels. These results are consistent with recent studies examining patients who had progressed on prior CDK4/6 inhibitor treatment alone and were subsequently treated with abemaciclib [12,20,29]. Similarly, the randomized MAINTAIN clinical trial demonstrated improved PFS in patients treated with ribociclib plus fulvestrant after progression on CDK4/6 inhibitors [47]. Notably, in these studies, most patients received prior palbociclib (n = 103, 87%). In these analyses, a subset of patients continued to derive clinical benefit from alternative CDK4/6 inhibitors, following progression on prior CDK4/6 inhibitor therapy. Additionally, a recent preclinical study [19] demonstrated that HR+, HER2− breast cancer models resistant to high-dose palbociclib also continued to derive benefit from treatment with abemaciclib, though abemaciclib-resistant models did not benefit from palbociclib treatment. While this study is in overall agreement with our observations, the authors indicate that the CDK4/6 inhibitor doses used were not clinically relevant (leading to potential off-target effects) and inconsistent with the clinical setting since no ET was used to develop the resistant models [20].

It is well established that the efficacy of CDK4/6i is achieved largely through the inhibition of Rb phosphorylation [48]. The inability of CDK4/6i to regulate Rb phosphorylation represents a canonical biomarker of resistance to CDK4/6i therapy [48,49]. Supporting these reports, we demonstrated that high, non-clinically relevant concentrations of palbociclib were required to decrease the pRb protein levels in ATxR models (Figure 2A). In contrast, abemaciclib + fulvestrant continued to robustly inhibit Rb phosphorylation in PTxR cells. These results suggest that (1) abemaciclib potently inhibits CDK4/6 and Rb phosphorylation following progression on palbociclib + ET and (2) progression towards CDK4/6i resistance is CDK4/6 inhibitor-dependent.

To further characterize our resistant models, we examined the composition of the CDK4/6–cyclin D1–p21/p27 complexes. Prior reports have demonstrated that the composition of the complex and the overall expression of CDK4 and CDK6 represent additional predictive factors of CDK4/6i response [16,18,30]. In PTxR cells, we observed greater CDK4-p21 interaction compared to ATxR models, consistent with previous descriptions of CDK4/6i sensitivity. In ATxR cells, the CDK6 levels were enriched, relative to PTxR models, and reported as an indicator of resistance to CDK4/6i.

Overall, the baseline protein expression revealed that resistance to abemaciclib and palbociclib + ET was consistent with previous molecular descriptions of both CDK4/6 inhibitor and tamoxifen resistance [14,50]. We observed the continued protein expression of ERα and increased pERK1/2 expression in ATxR and PTxR cells. Rb and pRb protein expressions were decreased and CDK6 expression was increased compared to the control. These models demonstrate both the bypass of the CDK4/6–cyclin D–Rb signaling axis [45] and the loss of dependence of ERα for ligand-mediated signaling [14], respectively. Although baseline ATxR and PTxR cells showed similar molecular characteristics consistent with CDK4/6i and tamoxifen resistance, phenotypically, we observed differential cytostatic and cytotoxic responses following the therapeutic switch. In line with these observations, we show decreased cyclin D and FoxM1 expression in untreated PTxR cells compared to both control and ATxR cells. Furthermore, the magnitude of CDK6 and pERK1/2 expression increase was greater in ATxR cells, and, overall, CDK2 and CDK4 expression was greater in ATxR cells, compared to in PTxR cells.

In addition to baseline molecular differences following the gain of resistance to CDK4/6i + ET, our results also demonstrated distinct treatment effects after the therapeutic switch linked to cell cycle regulation and survival ability. Recent reports have described the role of FoxM1 as a master regulator of cellular senescence and apoptosis [51]. In our resistant cell models, we observed decreased FoxM1 expression in PTxR cells at baseline and with the therapeutic switch, compared to in ATxR cells (baseline and therapeutic switch). These results support our observation that PTxR cells treated with abemaciclib + ET have decreased cellular proliferation and colony formation ability. Additionally, the treatment of ATxR cells with palbociclib + ET increased Rb/pRb and cyclin A protein expression, further validating the increased colony formation ability and, potentially, the magnitude of Ki-67 level changes, compared to PTxR cells at baseline and following the therapeutic switch. In conjunction with the overall increase in CDK6 expression observed in resistant cell lines, the magnitude of increased CDK6 expression was greater in ATxR cells, compared to in PTxR cells. These data are consistent with recent reports describing the role and composition of the CDK4/6-p21 and p27 complex as a biomarker of CD4/6 inhibitor response [30,52].

In support of our results, RNAseq analysis confirmed both tamoxifen- and endocrine-resistant gene signatures in the ATxR and PTxR cell models. Pathways related to cell cycle regulation and apoptosis were also differentially expressed following the therapeutic switch. The E2F pathway, a regulator of CDK expression and an oncogenic driver of Rb signaling [53], was upregulated in ATxR cells following treatment with palbociclib + ET and downregulated in PTxR cells when treated with abemaciclib plus ET. Genes related to Rb signaling also demonstrated an expression profile consistent with decreased cell proliferation in PTxR cells following treatment with abemaciclib + ET. Previous studies have suggested that the bypass of CDK4/6-mediated Rb regulation through receptor tyrosine kinases may drive resistance to CDK4/6 inhibitors [54,55]. Consistent with these reports, we observed the increased expression of EGFR-related genes following the development of resistance to abemaciclib plus tamoxifen and palbociclib plus tamoxifen. Given the clinical relevance of EGFR inhibitors [56,57], further studies are needed to explore the role of EGFR in mediating resistance to CDK4/6i + ET. Additionally, genes related to androgen response, epigenetic regulation, reactive oxygen species (ROS) response, fatty acid regulation, and RAS signaling were also differentially regulated dependent on the CDK4/6 inhibitor used to drive resistance. These findings suggest potential rational therapeutic approaches to overcome resistance to CDK4/6i + ET [31,35,45,58,59,60].

In summary, our results describe for the first time the phenotypic and molecular characteristics of early-onset resistance to CDK4/6i + ET in vitro. Palbociclib plus tamoxifen-resistant cells continued to show sensitivity to treatment with abemaciclib + ET (tamoxifen or fulvestrant), and the composition of the CDK4/6-p21 and p27 complex correlated with the treatment response. Overall, our results suggest CDK4/6i-dependent mechanisms of resistance that converge to drive and maintain downstream Rb and EGFR expression. The characterization of these cells and models of resistance post CDK4/6 inhibitor + ET treatment highlight the potential for novel therapeutic approaches to overcome resistance to CDK4/6i + ET in HR+, HER2− breast cancer.

## 4. Materials and Methods

### 4.1. Cell Line Generation and Culture Conditions

The T47D cell line was obtained from the American Type Culture Collection (ATCC #HTB-133, Manassas, VA, USA 30–4500 K). Cells were cultured according to ATCC recommendations for fewer than ten passages. For T47D Geminin cell line generation (conducted in collaboration with Dr. Marcos Malumbres’s group at the CNIO, Madrid, Spain), T47D cells were transduced with lentiviral solutions: the CSII-EF-MCS vector encoding mVenus-hGem(1/110) [61] was co-transfected with the packaging plasmids pMDLg/pRRE (Addgene #12251, Watertown, MA, USA) and pRSV-Rev (Addgene #12253) and the VSV-G envelope-expressing plasmid (Addgene #12259) into HEK293T cells (ATCC #CRL-3216, Manassas, VA, USA). Stable transformants were established by Venus-positive sorting.

### 4.2. Chemicals

Except when specified differently, all preclinical information detailed in this document was derived utilizing the methanesulfonate salt for both abemaciclib and palbociclib compounds and the succinate salt for ribociclib. Full chemical names have been previously published [62]. 4-hydroxytamoxifen and fulvestrant were purchased from Sigma-Aldrich (Merck, Darmstadt, Germany), and they were used at concentrations broadly accepted as efficacious.

### 4.3. Determination of Clinically Relevant Drug Concentrations

Free-drug concentrations used in cellular studies were determined as indicated in Supplemental Table S1. Unbound clinically relevant exposures of CDK4/6 inhibitors, human plasma fraction unbound, and cell culture fraction unbound for CDK4/6 inhibitor values were used.

### 4.4. Flow Cytometry Assays

#### 4.4.1. Geminin Analysis

In total, 100,000 cells were plated in a 6-well plate, and sequential treatments were conducted according to the instructions provided in Supplemental Figure S2. The cells underwent a single wash with PBS 1X and were trypsinized. Subsequently, the suspended cells were centrifuged in a tabletop centrifuge at 6000 rpm for 4 min. The resulting pellet was then resuspended in 4% PFA and fixed for 20 min at room temperature. After fixation, the cells were washed with PBS 1X, and the analysis of Geminin–Venus % was carried out using a blue 488nM laser (Filter FITC/Venus 525/50) (MACS Quant Analyzer 10) (Miltenyi, Bergisch Gladbach, Germany).

#### 4.4.2. Ki-67 Analysis

In a 6-well plate, 100,000 cells underwent sequential treatments following Supplemental Figure S2. After a single PBS 1X wash and trypsinization, the suspended cells were centrifuged at 6000 rpm for 4 min. The resulting cell pellet was resuspended in ice-cold 70% ethanol and fixed for at least 1 h at −20 °C. Post fixation, a PBS 1X wash was performed, and the cells were stained with Ki-67 (130-120-416, Miltenyi, Bergisch Gladbach, Germany) (1:50) for 20 min at room temperature. Subsequently, another PBS 1X wash was carried out, and the analysis of Ki67% utilized a Vio 667nM laser (APC channel) (MACS Quant Analyzer 10) (Miltenyi, Bergisch Gladbach, Germany).

#### 4.4.3. Annexin V-DAPI Analysis

In total, 25,000 cells per well were plated in 6-well plates; the cells were treated following the instructions in Supplemental Figure S2. Nine days post treatment, media containing floating cells were combined with cells harvested using accutase (A6964, Merck, Darmstadt, Germany). After centrifugation, the pellets were resuspended in 50 μL Annexin buffer 1× (130-092-820, Miltenyi) with antibody at a 1:50 ratio (130-118-363, Miltenyi) for 15 min at room temperature in the dark. Mitomycin C (200 nM) (Merck, Darmstadt, Germany) served as a positive control for apoptosis. Samples underwent a wash with 500 μL Annexin buffer 1×, were centrifuged for 4 min at 1200 rpm, and were then resuspended in 100 μL Annexin buffer 1× with 1:100 DAPI staining solution (Miltenyi 130-111-570). The stained cells were analyzed using VioBlue (V1) laser for DAPI and PE laser (B2) for Annexin V PE, with data analysis and interpretation following the manufacturer’s protocol.

The MACS Quant Analyzer 10 cytometer (Miltenyi, Bergisch Gladbach, Germany) was employed for all analyses, and FCS files were processed using Flow Jo v10.6.0.

### 4.5. Colony Formation Assay

After seeding 10,000 cells and subjecting them to a 14-day treatment in triplicate, colonies were fixed with 100% methanol for 5 min at room temperature and stained with 0.1% crystal violet for 1–2 min. The quantification of the colony area was conducted using ImageJ software (version 1.47n) (National Institute of Health, Bethesda, MD, USA) with the colony area plug-in. Statistical analysis was carried out relative to the resistant control cell line.

### 4.6. Dose–Response Assay

In total, 100,000 (Geminin analyses) or 300,000 (Biomarker analyses) cells were plated per well in 6 multi-well plates, and sequential treatments were performed over two doubling times. PTxR and ATxR were switched to abemaciclib and palbociclib, respectively, in serial dilutions of 1:3, starting at 20 µM plus fulvestrant at a fixed concentration of 4 nM. Geminin and Jess protein analyses were performed as described in Section 4.

### 4.7. Western Blot and Jess Simple Western^TM^ Assays

Cells were rinsed twice with ice-cold PBS, then lysed using ice-cold NP40-based lysis buffer (140 mM NaCl, 10 mM EDTA, 10% glycerol, 1% NP-40, 20 mM Tris, pH 8.0), supplemented with cOmplete mini protease inhibitor cocktail and PhosSTOP phosphatase inhibitors (Roche, Merck, Darmstadt, Germany). After scraping cells from the flasks, samples were centrifuged at 12,500 rpm at 4 °C, and the resulting supernatant was transferred to new tubes.

Western blot analysis was performed following our established protocol [21]. Jess protein analyses adhered to the manufacturer’s guidelines, with prior internal optimization of primary antibodies. For the Jess Simple Western™ instrument (ProteinSimple^®^, Bio-Techne, Minneapolis, MN, USA), lysates were diluted to specific total protein concentrations in 1x fluorescent master mix (EZ standard pack I; PS-ST01EZ-8, Biotechne), and 3 µL was added per well. Primary antibodies were diluted in antibody diluent 2 (042-203, Bio-Techne). Secondary antibodies (anti-rabbit HRP) and enhanced chemiluminescence (ECL) reagents were applied as per the kit’s instructions (anti-rabbit and anti-mouse detection module chemiluminescence; DM-001 and DM-002, Bio-Techne). The antibody diluent, washing buffer, plates, and capillary cartridges were sourced from the 12–230 kDa separation module (SM-W001, Bio-Techne).

A protein normalization assay (AM-PN01, Bio-Techne) was employed to ensure consistent protein content across all samples. Jess Simple Western™ data were analyzed using Compass for Simple Western software (version 6.3.0), relying on high dynamic range 4.0 images and Gaussian fit for peak detection and area analysis.

The primary antibodies used were as follows: ERα (RM-9101-S1, Thermo Fisher Scientific; Waltham, MA, USA), pRb Ser780 (173289, Abcam; Cambridge, UK), Rb (554136, BD Pharmingen; Franklin Lakes, NJ, USA), cyclin D (2978, Cell Signaling; Danvers, MS, USA), CDK4 (12790, Cell Signaling), CDK6 (124821, Abcam), cyclin A (C4710, Merck), CDK2 (2546, Cell Signaling), pERK1/2 (9101; Thr202/Tyr204, Cell Signaling), ERK1/2 (9102, Cell Signaling), FoxM1 (180710, Abcam), p21 (2947, Cell Signaling), p27 (3686, Cell Signaling), Topoisomerase IIα (12286, Cell Signaling), Mcl-1 (5453, Cell Signaling), Phospho-cdc2 (Tyr15; 9111, Cell Signaling), Wee1 (13084, Cell Signaling), RNA-Pol II CTD repeat YSPTSPS (phosphor S2; 5095; Abcam), pHistone H3 (Ser 10, 06-570, Merck), vinculin (MAB3574, Merck), and α-tubulin (MABT205, Merck). The secondary antibodies used in the conventional Western blot were goat anti-rabbit (P0448, Dako, Glostrup, DK) and goat anti-mouse (Dako; P0447).

### 4.8. Co-Immunoprecipitation

Sixteen million cells per condition were seeded and treated as outlined in Supplemental Figure S2. Ice-cold NP40-based lysis buffer was used to prepare total cell lysates. Immunoprecipitations (IPs) were conducted with protein A agarose (Roche) following manufacturing instructions. Antibodies for immunoprecipitations were as follows: CDK4 (226474, Abcam), CDK6 (124821, Abcam), and CDK2 (2546, Cell Signaling). The mixture was incubated for 5 h on a rocking platform at 4 °C. Immune complexes were recovered by a brief centrifugation, followed by three washes with 1 mL of cold lysis buffer. Dilution mix 1× (sample buffer 0.1× diluted with 5× fluorescent master mix, Protein Simple, Bio-techne) was added to the beads and boiled for 5 min.

### 4.9. RNAseq

RNAseq was performed on an Illumina NovaSeq 6000 using the Illumina TruSeq Stranded mRNA Library Prep Kit with paired-end sequencing, a read length of 75 bp, and a targeted read depth of 80–100 M reads/sample. Raw FastQ files were quality- and adapter-trimmed using cutadapt (cutadapt-1.9.1) and aligned using GSNAP (v2013-11-27, command line parameters -B 5 -A sam -N 1 -t 8 -s splicesites --quality-protocol=sanger --gunzip --sam-multiple-primaries --maxsearch=1000 --npaths=100) to build 37.p5 of the human genome. Read counts were generated against exons annotated in NCBI gene models (NCBI h37.p13 annotation) using a custom Perl script, then summarized at the gene level as the log2 of mean exon reads. The raw counts were upper-quantile-normalized and VST-transformed. The batch effect was removed before the downstream analysis, using the batchQC_fsva_adjusted function from the R package batchQC.

The gene set (pathway) definitions were either generated from the KEGG official website or downloaded from the Molecular Signature Database (MsigDB, http://www.broadinstitute.org/gsea/msigdb/, accessed on 19 July 2021). Multiple single-sample pathway analysis algorithms, parameter gene set enrichment analysis (PGSEA; ref. [63]), and multiple other single-sample gene set enrichment methods implemented in the gene set variation analysis (GSVA) R package [64], concretely z-score, plage, and ssGSEA, were used to generate enrichment scores for each pathway within each sample using the control cell lines (for ATxR cell line using abemaciclib/tamoxifen treatment; for PTxR cell line using palbociclib/tamoxifen treatment) as references. A moderated *t*-statistic implemented in the limma package was used to identify gene set enrichment scores that could discriminate between groups.

### 4.10. Statistical Analysis

All data, unless noted otherwise, are represented as the mean value ± SEM of at least three biological replicates. For biological assays (Western blot analysis, Jess analysis, Annexin V, colony formation assay, %GEM+, %Ki-67), a two-tailed, unpaired Student’s *t*-test was used.

## Data Availability

Data are contained within this article.

## Supplementary Materials

The supporting information can be downloaded at https://www.mdpi.com/article/10.3390/ijms26062643/s1. References [24,65,66,67,68,69,70] are cited in the Supplementary Materials.

## Abbreviations

AR	Androgen response
ATxR	Abemaciclib + tamoxifen resistant
CDK4/6	Cyclin-dependent kinase 4 and 6
CDK4/6i	CDK4/6 inhibitor
ET	Endocrine therapy (tamoxifen, fulvestrant)
GEM+	Geminin positive
HER2	Human epidermal growth factor receptor 2 negative
HR+	Hormone receptor positive
PTxR	Palbociclib + tamoxifen resistant
Tamoxifen	4-hydroxytamoxifen; 4-OH-tamoxifen
