# Supplementary material for: Models of Early Resistance to CDK4/6 Inhibitors Unveil Potential Therapeutic Treatment Sequencing
Source: Int J Mol Sci. 2025 Mar 14;26(6):2643. doi: 10.3390/ijms26062643 (PMC11942273; doi:10.3390/ijms26062643)
Supplement: Supplementary file 1 [file ijms-26-02643-s001.zip › ijms-3486418-supplementary.pdf]

Supplemental Table S1. Determination of cellular CDK4/6 inhibitor concentrations that would approximate clinically relevant exposures

CDK4/6 Inhibitor	Clinical Dose (mg)	Clinical Exposure Cmax (ng/mL)	Clinical Exposure Cmax (nM)	Human plasma fraction unbound, hFu pl (d)	Unbound clinical exposure Cmax,u (nM)	Measure cell media Fu 30% FBS	Calculated cell media Fu 10% FBS (e)	Clinical Cmax,u corrected for 10% FBS Fu (nM)	Concentration used in this study (nM)
**Abemaciclib**	150 mg bid	498 (a)	984	0.027	26	0.279	0.537	48	**40**
**Palbociclib**	125 mg qd	86 (b)	192	0.15	29	0.54	0.796	36	**40**
**Ribociclib**	600 mg qd	1820 (c)	4188	0.287	1202	0.682	0.877	1371	**1300**

(a) Abemaciclib clinical Cmax was taken from phase 1 JPBA study (1) (2) and doubled as it is estimated equal contribution to pharmacology from 3 equipotent and active metabolites (M2, M18 and M20) (3) (4) (2)
Rest of the calculations for abemaciclib concentrations include this assumption
(b) Palbociclib clinical Cmax was taken from phase 1 dose escalation study (4)(2)
(c) Ribociclib clinical Cmax was taken from FDA Review of clinical pharmacology (NDA documentation). See (5)
(d) Determined internally at Eli Lilly. See (6)
(e) Calculated from 30% FBS cell media Fu using conversion equation described in reference (7). See below.

$$\mathrm{fu}_2 = \frac{1}{\frac{C_2}{C_1}\left(\frac{1-\mathrm{fu}_1}{\mathrm{fu}_1}\right)+1}$$

References:

1. Patnaik A, Rosen LS, Tolaney SM, et al. Efficacy and safety of abemaciclib, an inhibitor of CDK4 and CDK6, for patients with breast cancer, non-small cell lung cancer, and other solid tumors. Cancer Discovery 2016, 6, 740–53. https://doi.org/10.1158/2159-8290.CD-16-0095.
2. Groenland, SL, Martínez-Chávez, A, van Dongen, MGJ, et al. Clinical Pharmacokinetics and Pharmacodynamics of the Cyclin-Dependent Kinase 4 and 6 Inhibitors Palbociclib, Ribociclib, and Abemaciclib. Clin. Pharmacokinetics 2020, 59, 1501–1520. https://doi.org/10.1007/s40262-020-00930-x
3. Turner K, Chappell J, Kulanthaivel P, et al. Food effect on the pharmacokinetics of 200-mg abemaciclib in healthy subject [abstract]. Proceedings of the 107th Annual Meeting of the American Association for Cancer Research; 2016 Apr 16-20; New Orleans (LA). Cancer Res. 2016, 76(14 Suppl.): abstract no. CT152.
4. Flaherty KT, LoRusso PM, DeMichelle A, et al. Phase I, Dose-Escalation Trial of the Oral Cyclin-Dependent Kinase 4/6 Inhibitor PD 0332991, Administered Using a 21-Day Schedule in Patients with Advanced Cancer. Clin. Cancer Res. 2012; 18(2), 568-576. https://doi.org/10.1158/1078-0432.CCR-11-0509
5. US Food and Drug Administration. Center for Drug Evaluation and Research. Clinical pharmacology review ribociclib. 2018. https://www.accessdata.fda.gov/drugsatfda_docs/nda/2017/209092Orig1s000ChemR.pdf
6. Raub TJ, Wishart GN, Kulanthaivel P, Staton BA, et al. Brain Exposure of Two Selective Dual CDK4 and CDK6 Inhibitors and the Antitumor Activity of CDK4 and CDK6 Inhibition in Combination with Temozolomide in an Intracranial Glioblastoma Xenograft. Drug Metabolism and Disposition 2015, 43(9), 1360-1371. https://doi.org/10.1124/dmd.114.062745
7. Austin, RP, Barton, P, Cockroft, SL, et al. The Influence of Nonspecific Microsomal Binding on Apparent Intrinsic Clearance, and Its Prediction from Physicochemical Properties. Drug Metabolism and Disposition 2002, 30 (12), 1497-1503. https://doi.org/10.1124/dmd.30.12.1497

Supplemental Table S1. Determination of cellular CDK4/6 inhibitor concentrations that would approximate clinically relevant exposures

Footnote

From publicly available reports from dose escalation studies [25, 26] and FDA clinical pharmacology reviews, [27] human C_{max} values broadly accepted in the literature were selected [23]. Abemaciclib clinical exposure considered the presence of three active and equipotent metabolites (M2, M18 and M20) that have been characterized and described to circulate in combined similar concentration to their parent drug. Human plasma fraction unbound was determined internally at Eli Lilly and Company to derive the relevant unbound clinical C_{max} for the three molecules.
Subsequently, 10% FBS containing media fraction unbound (Fu) was calculated from available 30% FBS containing media Fu. Correcting the unbound C_{max} with the 10% FBS media Fu allowed us to determine the relevant concentration to be added in conventional tumor cell culture that would correspond to free drug concentrations in cells that best represent the unbound clinical C_{max}.

The IC_{90} values were corrected to account for the fraction unbound (Fu) for comparison purposes. This adjustment ensures that we are considering only the portion of each compound that is not bound to proteins, as this unbound fraction is expected to have the pharmacological effect.

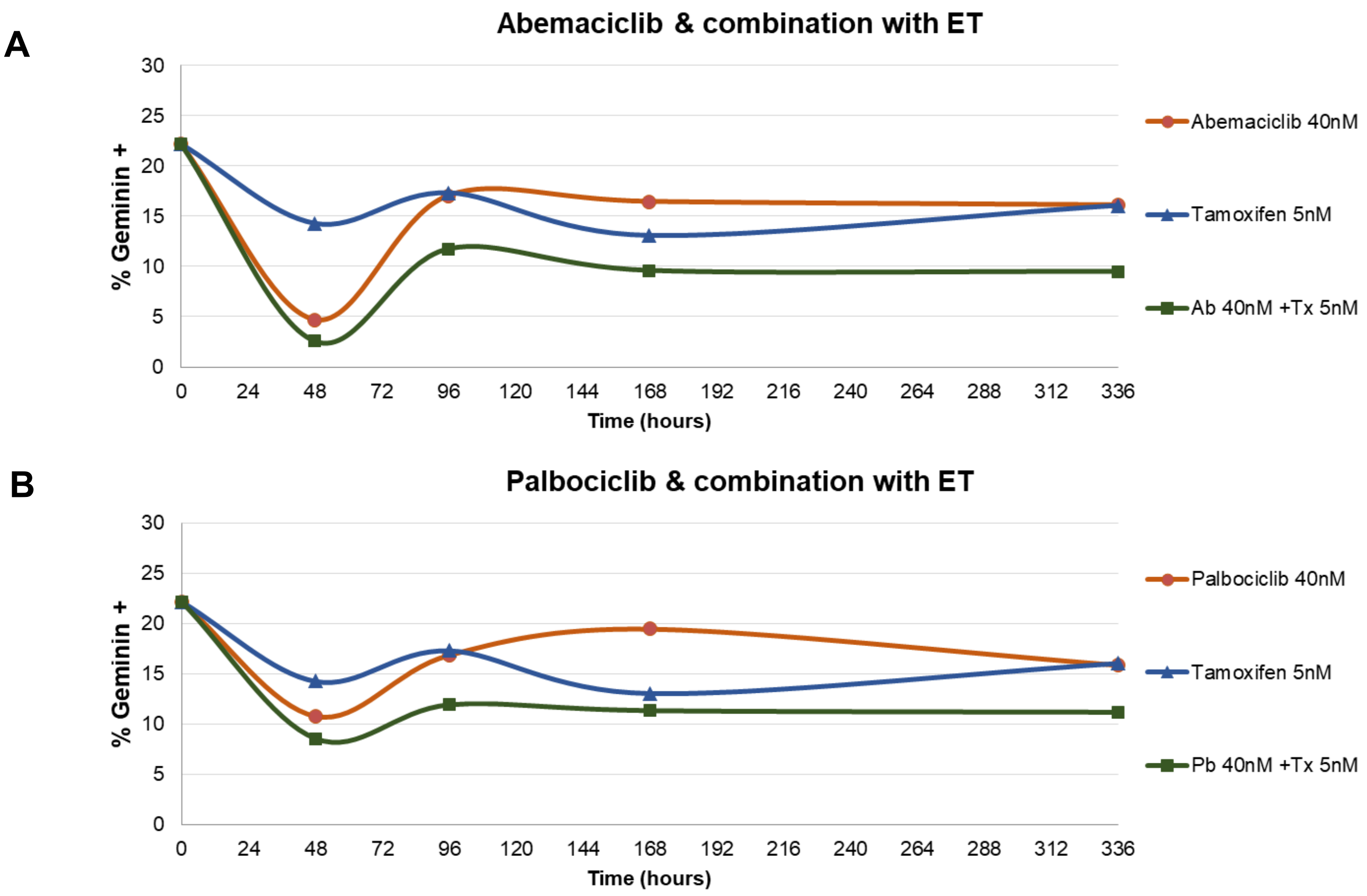

Supplemental Figure S1. Time-course for selection of incubation time and drug concentration for generation of resistant cell lines. T47D Geminin cells were treated with CDK4/6i (abemaciclib or palbociclib) (40nM), tamoxifen (5nM) and fulvestrant (4nM) as single agents or in combination at 48, 96, 168 and 336 hours and geminin percentage was analyzed by flow cytometry.

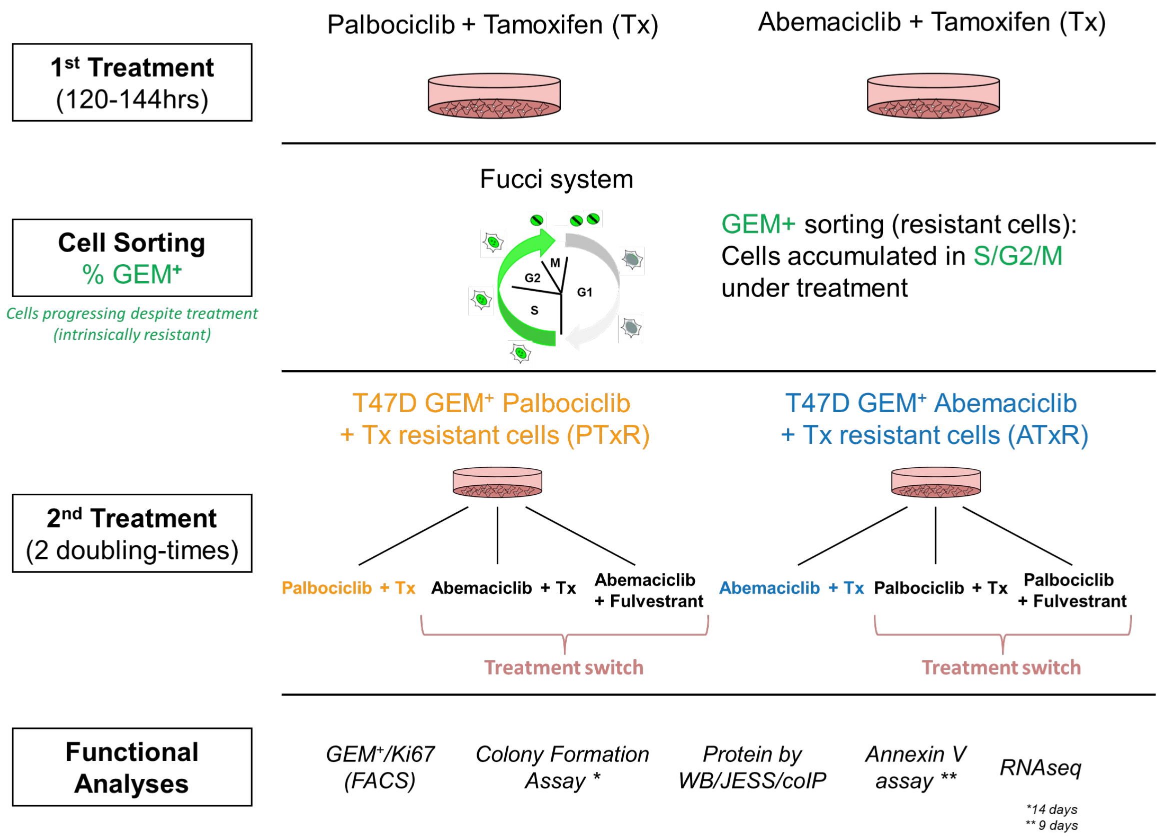

Supplemental Figure S2. Experimental design of sequential treatments T47D Geminin parental cells were treated with CDK4/6i 40nM (abemaciclib or palbociclib) in combination with tamoxifen (5nM) for 120-144hrs and sorted for resistant cells defined as geminin positive (GEM+), a marker of S/G2/M cell cycle phases. To confirm the resistant phenotype, cell lines were treated with tamoxifen plus the CDK4/6 inhibitor used to drive resistance. To understand if sequential CDK4/6i treatment is effective in controlling cell proliferation, cell lines were treated with the opposite CDK4/6 inhibitor plus ET (fulvestrant (4nM) or tamoxifen (5nM)).

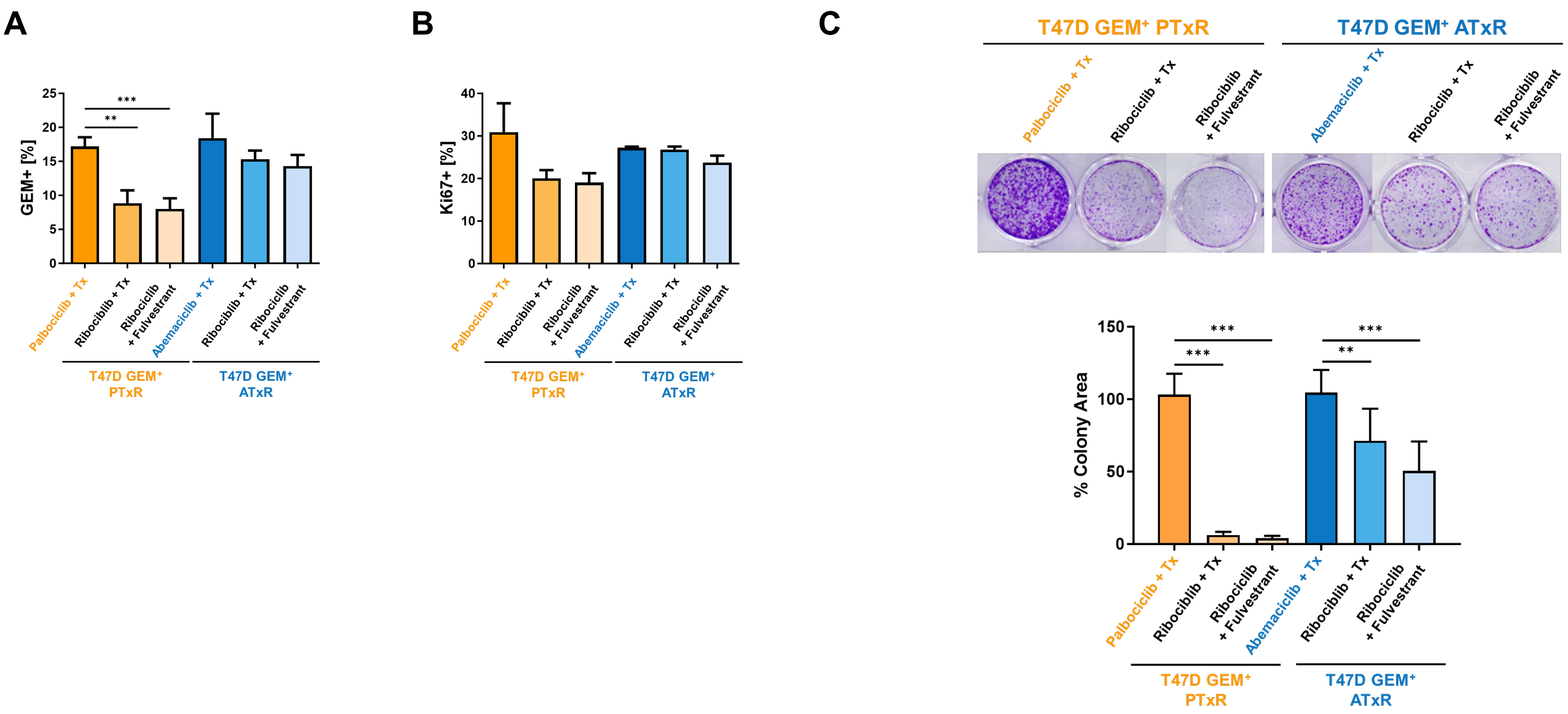

Supplemental Figure S3. Sequential treatments in T47D GEM+ PTxR and T47D GEM+ ATxR cells with Ribociclib + ET. A) GEM+ % and **B)** Ki67 % were measured in parallel to assess the proliferating fraction of a cell population (n=3, two-tailed, unpaired Student's t-test to resistant control line). **C)** Colony formation assay in T47D GEM+ PTxR and T47D GEM+ ATxR cell lines. 10000 cells/well were seeded and cultivated in the presence of Ribociclib (1.3uM)+ 4-OH-tamoxifen (5nM) and/or fulvestrant (4nM) , as well as vehicle control (DMSO/Methanol, data not shown) for 14 days. Colonies were fixed using 100% methanol for 5 minutes RT and stained with 0.1% crystal violet for 1-2 minutes. Colony area was quantified with Image J (n=3, two-tailed, unpaired Student's t-test to resistant control line). Representative image is shown from n=3.

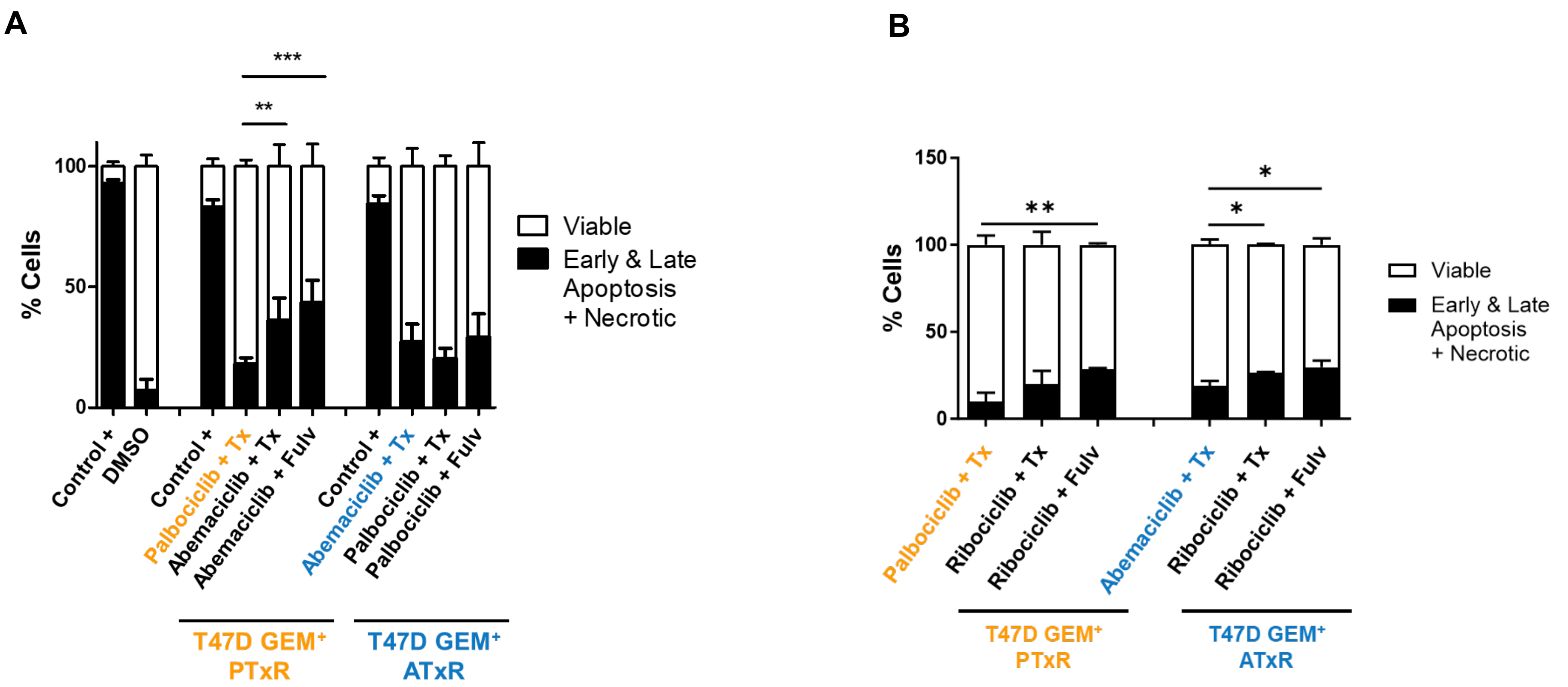

Supplemental Figure S4. Induction of apoptosis by the different sequential treatments was analysed in both resistant cell lines by flow cytometry at 9 days post-treatment. **A)** Switches from PTxR cells to Abemaciclib +ET and ATxR cells to Palbociclib + ET**. B)** Switches from ATxR and PTxR cells to Ribociclib + ET. Cells were harvested and incubated with Annexin V/DAPI. Annexin V PE single-positive cells were early apoptotic cells, Annexin V PE and DAPI double-positive cells were late apoptotic cells, and DAPI single-positive cells were dead cells. Mitomycin C was used as a positive control for apoptosis induction (n=3, two-tailed, unpaired Student's t-test to resistant control line).

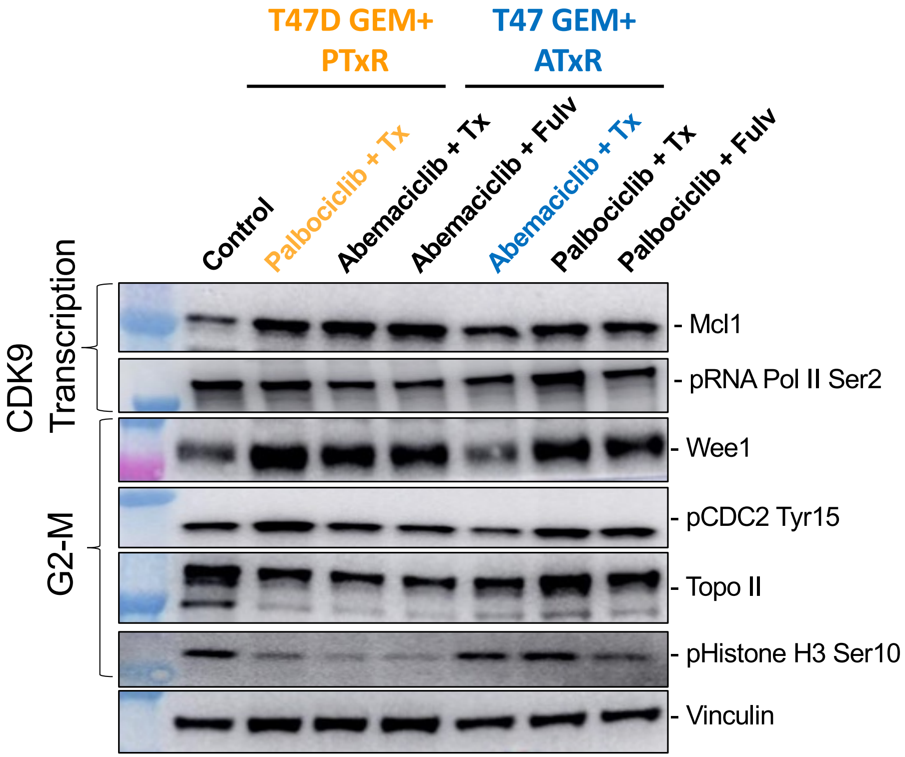

Supplemental Figure S5. Addressing literature-described off-targets effects for CDK4/6 inhibitors. Western blot images of proteins related to CDK9 transcription (Mcl1 and phospho RNA Polymerase II repeat YSPTSPS (phospho S2)) and G2-M cell cycle phases (Wee1, phospho CDC2 Tyr15, Topoisomerase II alpha and phospho Histone H3 Ser10) (n=2; representative images shown). Vinculin was assayed as a housekeeping gene.

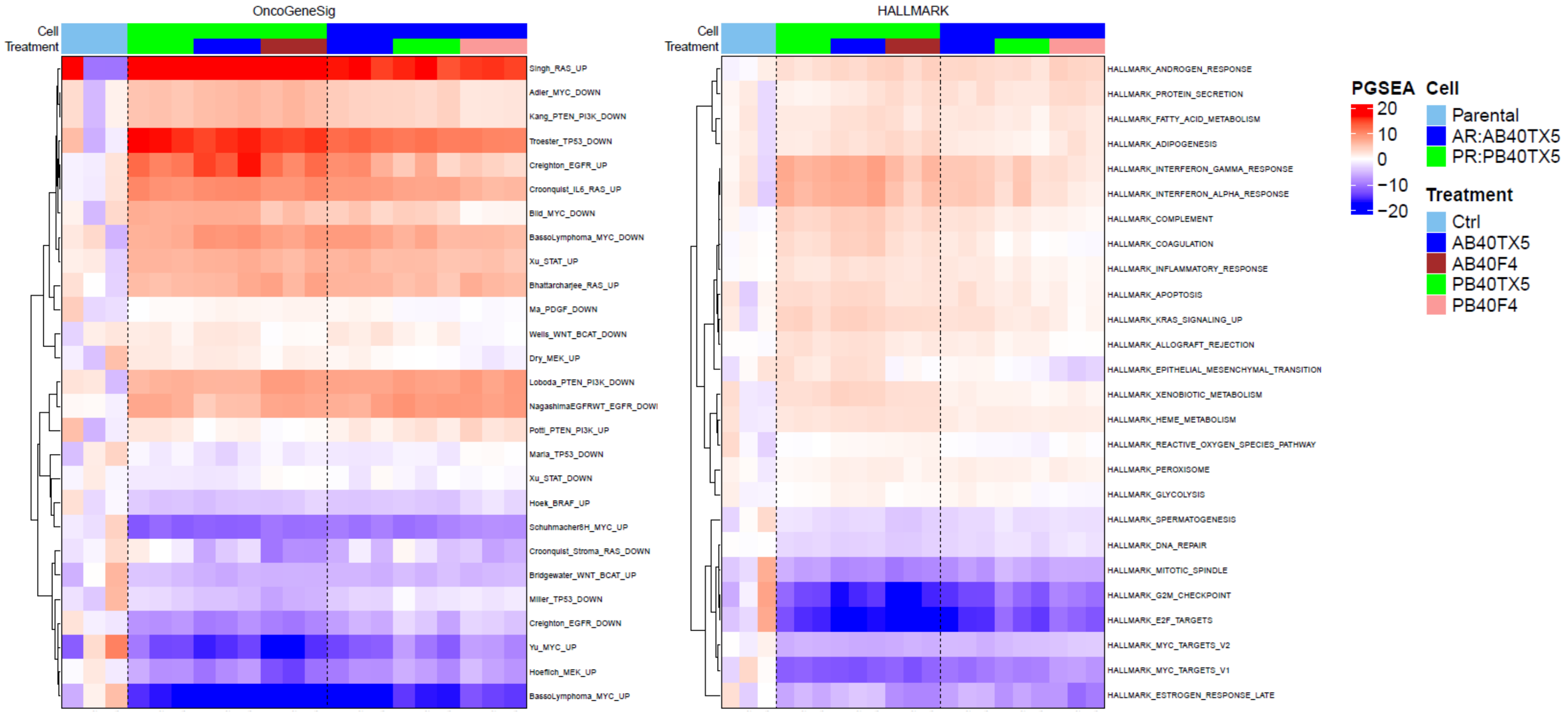

Supplemental figure S6. Heatmap for top changed HALLMARK and OncoGeneSig

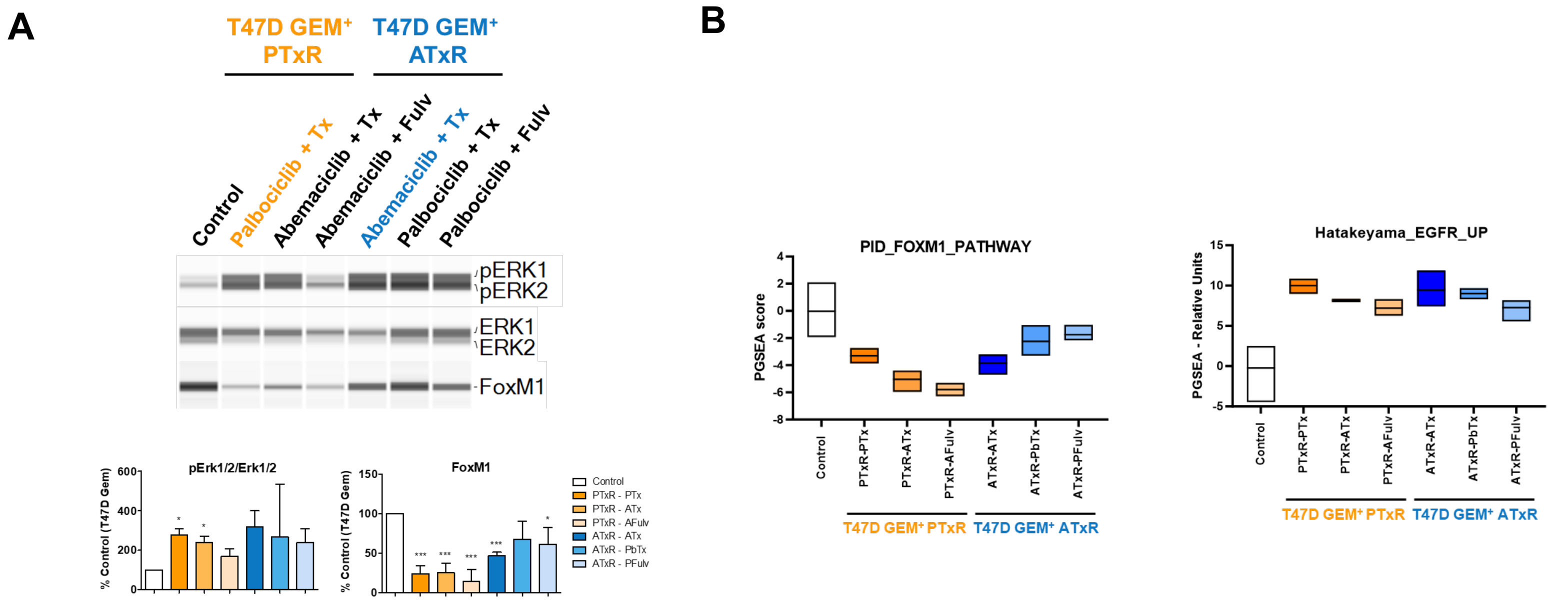

Supplemental Figure S7. Analyses of the protein levels of pErk1/2, Erk1/2 and FoxM1 and PGSEA of FOXM1 and EGFR in resistant PTxR and ATxR cell lines and drug switches. (A) Western blot-liked images of cell cycle marker panel and quantification of total peak area to determine changes in target protein expression, represented as % Control calculated to T47D Geminin parental cell line treated with DMSO/Methanol (n=3, two-tailed, unpaired Student's t-test to vehicle control). pErk1/2 was normalized to total Erk1/2. **(B)** PGSEA score for FoxM1 and EGFR pathways.
